# Analysis of codon usage bias of classical swine fever virus

**DOI:** 10.14202/vetworld.2021.1450-1458

**Published:** 2021-06-05

**Authors:** Sharanagouda S. Patil, Uma Bharathi Indrabalan, Kuralayanapalya Puttahonnappa Suresh, Bibek Ranjan Shome

**Affiliations:** ICAR-National Institute of Veterinary Epidemiology and Disease Informatics (NIVEDI), Yelahanka, Bengaluru, Karnataka, India

**Keywords:** classical swine fever virus, codon usage bias, India, nucleotide composition, synonymous codons

## Abstract

**Background and Aim:**

Classical swine fever (CSF), caused by CSF virus (CSFV), is a highly contagious disease in pigs causing 100% mortality in susceptible adult pigs and piglets. High mortality rate in pigs causes huge economic loss to pig farmers. CSFV has a positive-sense RNA genome of 12.3 kb in length flanked by untranslated regions at 5’ and 3’ end. The genome codes for a large polyprotein of 3900 amino acids coding for 11 viral proteins. The 1300 codons in the polyprotein are coded by different combinations of three nucleotides which help the infectious agent to evolve itself and adapt to the host environment. This study performed and employed various methods/techniques to estimate the changes occurring in the process of CSFV evolution by analyzing the codon usage pattern.

**Materials and Methods:**

The evolution of viruses is widely studied by analyzing their nucleotides and coding regions/codons using various methods. A total of 115 complete coding regions of CSFVs including one complete genome from our laboratory (MH734359) were included in this study and analysis was carried out using various methods in estimating codon usage bias and evolution. This study elaborates on the factors that influence the codon usage pattern.

**Results:**

The effective number of codons (ENC) and relative synonymous codon usage showed the presence of codon usage bias. The mononucleotide (A) has a higher frequency compared to the other mononucleotides (G, C, and T). The dinucleotides CG and CC are underrepresented and overrepresented. The codons CGT was underrepresented and AGG was overrepresented. The codon adaptation index value of 0.71 was obtained indicating that there is a similarity in the codon usage bias. The principal component analysis, ENC-plot, Neutrality plot, and Parity Rule 2 plot produced in this article indicate that the CSFV is influenced by the codon usage bias. The mutational pressure and natural selection are the important factors that influence the codon usage bias.

**Conclusion:**

The study provides useful information on the codon usage analysis of CSFV and may be utilized to understand the host adaptation to virus environment and its evolution. Further, such findings help in new gene discovery, design of primers/probes, design of transgenes, determination of the origin of species, prediction of gene expression level, and gene function of CSFV. To the best of our knowledge, this is the first study on codon usage bias involving such a large number of complete CSFVs including one sequence of CSFV from India.

## Introduction

Classical swine fever (CSF) is caused by an enveloped RNA virus belonging to the family *Flaviviridae* of genus *Pestivirus*. It was found that the classical swine fever virus (CSFV) is antigenically related to the other pestiviruses such as bovine viral diarrhea virus of cattle, and border disease virus of sheep. CSFV is a highly prevalent and endemic disease, usually found affecting the swine. The infected pigs develop few symptoms such as diarrhea, nausea, fever, hemorrhages, stagnation, and discoloration seen in legs, ears, and abdomen. They might also develop neurological disorders, reproductive disorders, and usually abortions [1-3].

The studies on analysis of codon usage pattern on CSFV are minimum or less. CSF is a very serious contagious disease found infecting different places around the world. The codon usage analysis is the most essential feature that plays a major role in biological evolution. The codon usage bias is found in the coding DNA, with difference in the frequencies of synonymous codons occurrences [4,5]. The synonymous codon is those which codes for the same amino acid, except for the codons that encode methionine and tryptophan. Some of the synonymous codons usage varies in different species, which is not random [5-7]. Natural selection, nucleotide base content, genetic mutation, and drift are some of the factors that are closely related to the codon bias in the molecular evolution of the agent/organisms. The codon usage experienced during the process of molecular evolution, is efficient in changing the production of proteins and mutations in the genes [5-9]. Therefore, codon usage analysis provides details on how it affects the evolution pattern, environmental adaptation, response to the immune system, and virus survival among the hosts and virus [7,10]. Further, analysis of codon usage bias is important in understanding the molecular biology, genetics, and genome evolution, it also helps in new gene discovery, design of primers, design of transgenes, determining the origin of species, and prediction of gene expression level and gene function.

Thus, the analysis on codon usage bias helps in obtaining an in-depth knowledge of mutations that leads to evolutionary changes and also to understand the changes in the viral adaptations. This study performed and employed various methods/techniques to estimate the changes occurring in the process of CSFV evolution by analyzing the codon usage pattern.

## Materials and Methods

### Ethical approval

Ethical approval is not applicable since the study used the data available in the public domain.

### Study period and location

A total 115 complete CSFV sequences obtained from 1977 to 2019 from the GenBank (NCBI) were used in the study. The sequences were derived from 3 continents viz., Asia, Europe, and North America (Supplementary data can be available from the corresponding author).

### Sequence data retrieval

One complete CSFV genome sequence from our laboratory (MH734359) [11], along with a total of 114 coding sequences (CDS) of CSFV from 20 different countries were retrieved from the GenBank database, National Center for Biotechnology Information (NCBI) (https://www.ncbi.nlm.nih.gov/nucleotide/). All the strains representing six subtypes (1.1, 1.2, 2.1, 2.2, 2.3, and 3.2) were used in this study and sequences with >99% homogeneity were excluded (Supplementary data can be available from the corresponding author).

### Nucleotide composition analysis

The whole-genome sequences of CSFV were aligned and edited using MEGA X (https://www.megasoftware.net/). The mononucleotide frequencies (A, G, C, and T), the contents of GC at first, second, and third codon positions (GC_1_, GC_2_, and GC_3_), and GC_12_ (mean of GC_1_ and GC_2_) were calculated using Seqinr library [12] in R software [13]. The frequencies of mononucleotides at the third position of synonymous codon (A_3_, G_3_, C_3_, and T_3_) were obtained from MEGA X [14]. The index GC_3_ (at synonymous third codon position) was used to calculate the fraction of GC nucleotides at the synonymous third codon position (excluding Met [Methionine], Trp [Tryptophan], and the termination codons) [15]. These nucleotide parameters were used for further analysis to obtain codon usage analysis.

### Effective number of codons (ENC) and ENC-plot analysis

The ENC values were used to enumerate the complete usage pattern of codon bias in the coding sequences (ORFs) and how it varies from the normal usage of synonymous codons. ENC is considered as an estimator of codon usage bias in ORF. The values of ENC ranges from 20 to 61, indicating that the values closer to 20 preferred to have stronger codon usage bias whereas values nearer or equal to 35 have moderate codon usage bias and values closer to 60 have weaker codon usage bias. The ENC value is usually estimated with the following mathematical formula [16,17]:


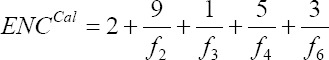



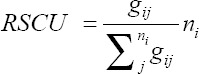


Where*, f_2_, f_3_, f_4_*, and *f_6_* stands for values of *f_i_* for i-fold degenerate amino acids and the coefficients 9, 1, 5, and 3 indicate the different classes of amino acid. The *f_i_* is estimated with the formula [17]:


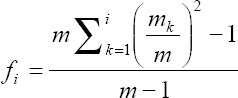


The total number of observed codons for the amino acid is represented as m, the observed number of the *k*th codon for the amino acid is represented as *m_k_*. The coRdon library [18] in R software [15] was used to estimate the ENC values.

The relationship between ENC and GC_3_ value is mostly used to know how the codon usage patterns are influenced by several factors such as mutation pressure and natural selection. Therefore, ENC-plot analyzes the relationship between ENC and GC_3_ values. In the ENC-plot, the ENC values for every GC_3_ values were calculated with the formula as follows:


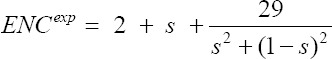


Where the GC_3_ values are denoted as *s*, and with the expected ENC values, a curve was produced. In the plot, if the observed ENC-GC_3_ values fall on the curve, it means that mutation was the main force acting on third position bases of codons whereas if observed ENC values fell considerably below the expected curve, it meant that selection was the main force driving codon usage bias [8-10]. If there is no natural selection, then evolution is mostly affected by mutational pressure. The codon usage would usually get affected by compositional parameters of the sequences. Therefore, the points are observed to fall on or near the expected ENC curve.

### Relative synonymous codon usage (RSCU) and principal component analysis (PCA)

The RSCU values of each codon in each gene were used to measure codon usage. The RSCU value is the ratio of observed frequency value to the expected frequency value of the synonymous codons [10]. The RSCU values were calculated with the following formula:


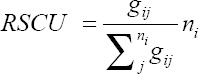


Where the observed number of the *i*th codon for the *j*th amino acid having *n_i_* synonymous codons. The RSCU values >1 represents codon abundance and have positive codon usage bias, whereas the RSCU values <1 represent less codon abundance and have negative codon usage bias. If the RSCU values are equal to 1, then there is no codon usage bias. Further, if the RSCU values >1.6 represents overrepresented codons and <0.6 represents underrepresented codons [7-10]. The RSCU values were estimated using the Seqinr library of R software [12,19]

PCA is a dimensionality reduction technique that is mostly used to obtain the relationship between variables (RSCU) and their components (codons). To analyze the variants and dominant patterns in the usage of codons on coding sequences in CSFV, the PCA [20] was performed on the RSCU values except for the three stop codons and the two sense non-synonymous codons ATG and TGG. The 59 RSCU values for each sequence with their codons were taken for the PCA. The analysis was done using factoextra library [21] in R software. The factors that influenced codon usage bias were effectually validated with the analysis of PCA.

### Codon adaptation index (CAI)

To measure the similarities in the usage of codon between the host and the virus, a CAI was performed. The CAI values range between 0 and 1; the higher CAI value indicates codon usage bias is higher and adaptive [22]. The CAI values were calculated using the DAMBE v7.2.1 software [23] with reference organism as *Sus scrofa* (pig). Those sequences with higher CAI values were chosen over the lower CAI values. It also indicates that the frequently used codons will preferably get adapted to their host [24].

### Neutral evolution analysis

The neutrality evolution plot represents the influence of mutation pressure and natural selection effects on the codon usage bias. The neutral evolution is analyzed by plotting the regression line with the synonymous codons values of GC_3_ against GC_12_ [25,26]. In this analysis, if the values are closer to one, they are statistically significant and the codon usage is mainly due to mutation pressure. If the slope is closer to zero, the selection is natural to codon usage bias. The linear regression analysis was performed using R software.

### Chargaff’s second parity rule (PR2) analysis

According to Chargaff’s PR2, mononucleotides A=T and G=C in the coding sequences indicate that there is no bias in the selection and mutation. To evaluate the effect of mutation and natural selection pressure on the codon usage pattern, the PR2 is plotted with AT bias at third codon position [A_3_/(A_3_+T_3_)] as ordinate against GC bias, at third codon position [G_3_/(G_3_+T_3_)] as abscissa and the origin at (0.5, 0.5) where A=T and G=C points lying have no bias with no affect towards mutation pressure and natural selection [10,27]. It is observed that the preference is toward purine than pyrimidine when the value is >0.5. Moreover, the mononucleotides base A is preferred over base T and base G is preferred over base C [28]. The bias resulting from mutations and natural selection helps us to measure the degree of deviance from PR2 [29].

### Dinucleotide abundance frequency analysis

Dinucleotide abundance frequency was performed to analyze the effect of dinucleotide frequencies on codon usage patterns. The frequencies of dinucleotides are considered overrepresented if the value is >1.23 and underrepresented if <0.78. The dinucleotide frequency is calculated with the formula as follows [30]:


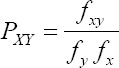


Where the frequency of nucleotides X and Y is denoted as *f_x_ and* *f_y_* respectively. The expected frequency of the dinucleotide XY is denoted as *f_y_f_x_* and the observed frequency of dinucleotide XY is denoted as *f_xy_* [9].

## Results

### Sequence data retrieval

In this study, a total of 115 CSFV coding sequences (complete genome), including one from our laboratory (MH734359) were downloaded from the GenBank database of NCBI (https://www.ncbi.nlm.nih.gov), with their accession numbers in FASTA format on October 21, 2020. In this study, all 115 CSFV coding sequences were included for the codon usage analysis.

### Nucleotide compositional analysis of CSFV

**Figure-1:**
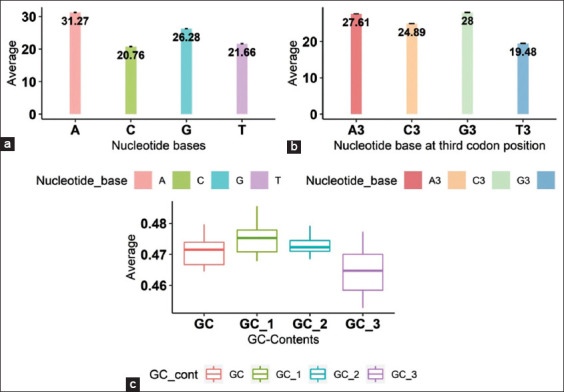
(a-c) Bar graphs showing nucleotide compositions of mononucleotides (A, T, G, C), Mononucleotides A_3_, C_3_, G_3_, and T_3_ at third codon site, and GC contents of classical swine fever virus.

The nucleotide content of the sequences was calculated, the frequencies of A, C, G, and T were 31.27%, 20.76%, 26.28%, and 21.66%, respectively, and the mean composition of nucleotide A is higher and nucleotide C is the least (Figure-1A). The codon composition at the third position G_3_, C_3_, A_3_, and T_3_, was 27.61%, 24.89%, 28.00%, and 19.48%, respectively, and the composition of A_3_ was found higher than the other nucleotides (Figure-1B). The mean compositions of GC, GC_1_, GC_2_, GC_3_, and GC_12_ were 0.470, 0.474838, 0.472834, 0.464195, and 0.473836, respectively (Figure-1C). GC_1_ and GC_2_ are higher and almost equal, whereas GC_3_ is low compared to GC_1_ and GC_2_ (Table-1).

**Table-1:** Frequencies of mononucleotides, GC contents, ENC and CAI of CSFV used in this study.

Mononucleotides	Frequency
A	0.31±0.04
C	0.20±0.04
G	0.26±0.04
T	0.21±0.04
A_3_	0.28±0.03
C_3_	0.24±0.03
G_3_	0.27±0.03
T_3_	0.19±0.03

**GC-contents**	**Frequency**

GC	0.47±0.003
GC_1_	0.47±0.004
GC_2_	0.47±0.002
GC_3_	0.46±0.006
GC_12_	0.47±0.002
ENC value	52.69±0.47
CAI value	0.71±0.003

A, C, G, and T denotes compositional frequency of A, C, G, and T. A_3,_ C_3_, G_3_, and T_3_ denotes compositional frequency of A_3_, C_3_, G_3_, and T_3_ at third codon site, GC_1_, GC_2,_ GC_3_ denotes the GC contents at first, second and third codon positions respectively. GC_12_ denotes mean of GC_1_ and GC_2_. ENC values represent the mean effective number of codons and CAI value represents the mean of Codon Adaptation Index of CSFV. CSFV=Classical swine fever virus, CAI=Codon adaptation index, ENC=Effective number of codons

### Effective number of codons (ENC) and ENC plot analysis of CSFV

The ENC is an essential component to evaluate the codon usage pattern and plays a very significant role in codon usage bias. In this study, the ENC values of CSFV coding sequences were ranging from 51.86 to 53.45, with 52.69 as the mean ENC value showing a low codon usage bias. These results indicate that all the ENC values of CSFV are very high, as every ENC value is usually >55. The codon usage bias in CSFV is high compared to other RNA viruses (Table-1).

**Figure-2:**
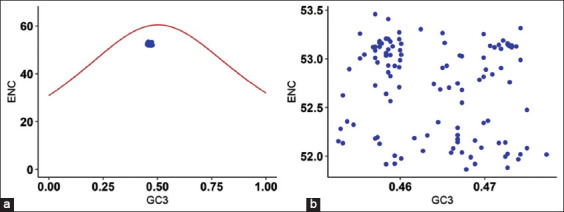
(a and b) Effective number of codons (ENC)-plot of classical swine fever virus (CSFV), the expected ENC curve plot represents ENC and GC_3_ values and scatter plot shows the relationship between ENC and GC_3_ values of all 115 CSFV sequences.

To analyze the usage of synonymous codons, the ENC values were plotted against GC_3_ values. The scatter plot shows the relationship between ENC and GC_3_ values which range between 51.86 and 53.45 of all 115 CSFV sequences (Figure-2B). In the ENC-plot it is seen that all the values fall inside and closer to the expected curve henceforth indicating that the selection pressure is influenced by codon usage bias in CSFV. These results indicate that the mutation in GC_3_ may also influence the codon usage bias in these sequences (Figure-2A).

### RSCU of CSFV

RSCU values are usually amino acid composition independent and are mainly used to compare the codon usage among the sequences. Among the 59 codons, 17 codons were mostly used. In these 17 codons, four (ATA, CCA, CAA, and TCA) codons were ending with A/T and 13 (GCC, TGC, GAC, GAG, TTC, GGG, CAC, AAG, CTG, AAC, AGG, and GTG) codons were ending with C/G. ATA (1.60), CTG (1.64), AGG (2.86), and AGA (2.59) were seen overrepresented (>1.6) and CGT (0.09), GCG (0.38), ACG (0.43), and CTT (0.45) were seen underrepresented (<0.6) (Table-2).

**Table-2:** RSCU values of 115 CSFV.

Amino acid	Codon	RSCU values	Amino acid	Codon	RSCU values
Phenylalanine	TTT	0.97±0.03	Serine	AGC	1.64±0.11
	TTC	1.06±0.07		AGT	0.45±0.05
Leucine	CTT	1.02±0.03		TCA	0.97±0.02
	CTC	0.93±0.07		TCC	1.15±0.07
	CTA	1.29±0.10		TCG	1.02±0.02
	CTG	1.30±0.05		TCT	0.84±0.07
	TTA	0.43±0.10	Threonine	ACA	1.36±0.05
	TTG	0.96±0.10		ACC	1.36±0.06
Isoleucine	ATA	2.59±0.09		ACG	0.38±0.04
	ATC	1.35±0.08		ACT	0.88±0.05
	ATT	2.86±0.10	Tyrosine	TAC	0.87±0.09
Valine	GTA	1.41±0.09		TAT	0.86±0.04
	GTC	1.60±0.05	Glutamine	CAA	1.39±0.05
	GTG	0.90±0.08		CAG	0.86±0.04
	GTT	0.48±0.11	Asparagine	AAC	0.97±0.05
Proline	CCA	1.11±0.03		AAT	0.86±0.07
	CCC	1.12±0.08	Cysteine	TGC	1.43±0.08
	CCG	0.88±0.03		TGT	0.72±0.11
	CCT	0.87±0.08	Histidine	CAC	1.18±0.04
Alanine	GCA	1.47±0.07		CAT	0.81±0.04
	GCC	0.91±0.06	Arginine	AGA	1.53±0.10
	GCG	0.64±0.08		AGG	0.73±0.07
	GCT	0.95±0.09		CGA	0.18±0.11
Glycine	GGA	0.12±0.04		CGC	0.76±0.09
	GGC	0.12±0.02		CGG	1.13±0.09
	GGG	0.19±0.04		CGT	0.86±0.09
	GGT	0.09±0.03	Aspartic acid	GAC	0.77±0.05
Lysine	AAA	1.09±0.08		GAT	1.11±0.04
	AAG	0.72±0.04	Glutamic acid	GAA	1.30±0.10
				GAG	0.88±0.04

59 codons with corresponding amino acids of all 115 coding sequences of CSFV sequences, the values in bold represents the overrepresented codons in CSFV. CSFV=Classical swine fever virus, RSCU=Relative synonymous codon usage

### Visualization of RSCU of CSFV

**Figure-3:**
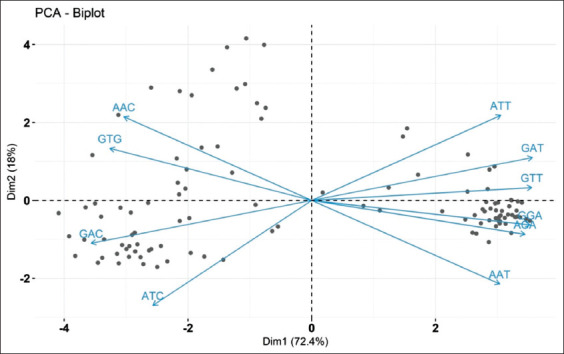
Principal component analysis (PCA) plot showing the deviations and similarity among the 59 codons of 115 classical swine fever virus sequences. PCA plot analysis showed the first principal component (72.4%) and second principal component (18%) of all the 59 synonymous codons variations in the relative synonymous codon usage values.

The PCA was performed with the RSCU values of CSFV. PCA plot analysis showed the first principal component (72.4%) and second principal component (18%) of all the 59 synonymous codons variations in the RSCU values (Table-2). Only the most represented codons among 59 codons have been considered as components. The codon usage pattern was influenced by the evolution in the RSCU analysis. The codons ending with A/T and G/C might influence the selection and mutation pressure (Figure-3).

### CAI of CSFV

To evaluate the impact of the virus in the host, an effective extent of codon usage bias in CSFV, the CAI was calculated using DAMBE v7.2.1 [23]. In this study, the average value of CAI in CSFV was found to be 0.71 and also falls between 0 and 1, indicating that the synonymous codons of CSFV are frequently used (Table-1). It evaluates the measure of natural selection and how the codon usage bias is influenced among the CSFV sequences.

### Neutral evolution analysis of CSFV

**Figure-4:**
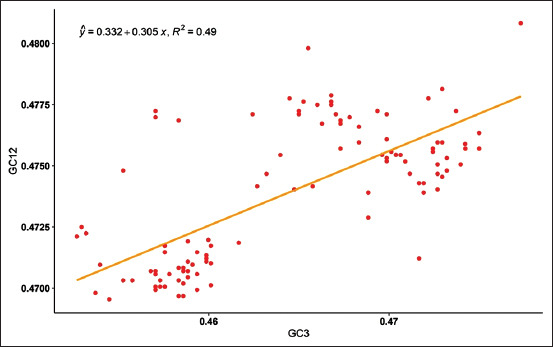
The neutrality plot analysis of classical swine fever virus (CSFV) sequences shows the correlation between the GC_3_ and GC_12_ and showing the influence of mutational bias in the CSFV.

The neutrality plot was analyzed by plotting the values of GC_3_ against GC_12_; the plot was significant (y= 0.332+0.305x, R^2^= 0.49) with p<0.05. The contents of GC_12_ and GC_3_ were varying slightly with an indication of low selection pressure; the codon usage pattern is influenced by GC contents of the nucleotides, and the natural selection contributed to the evolution of the codon usage pattern of CSFV (Figure-4).

### Chargaff’s second PR2 analysis of CSFV

**Figure-5:**
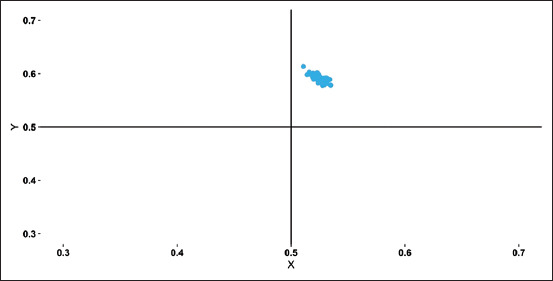
The PR2 plot was plotted using the obtained values with X= G3/(G3+C3) and Y=A3/(A3+T3) showing the mutation bias in classical swine fever virus.

To analyze the factors causing the codon usage bias in the CSFV, the PR2 bias, AT bias was plotted against GC bias, there was a minor deviation from the PR2 (A=T and C=G), whereas in the present study mononucleotide A was not equal to mononucleotide T and mononucleotide G was not equal to mononucleotide C in the third codon positions. In the PR2 plot, the distance between the values and the center indicates PR2 bias by its degree. Analysis revealed that AT and GC bias points were observed between 0.5 and 0.6, indicating lower bias (Figure-5). It was found that the mean AT bias was 0.52 and GC bias was 0.58. Since the values are >0.5, A and G (Purines) are preferred over T and C (Pyrimidines).

### Dinucleotide abundance frequency analysis of CSFV

**Figure-6:**
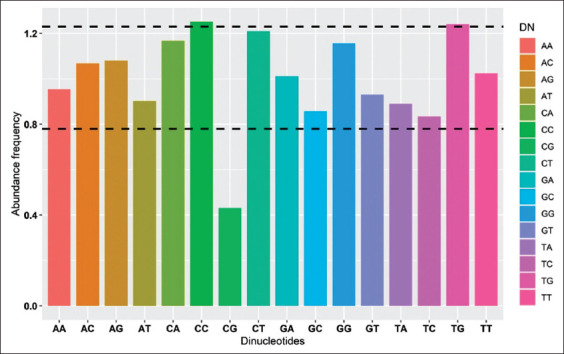
Dinucleotide abundance frequency of the classical swine fever virus (CSFV). The lines showing overrepresented and underrepresented values. The color variation represents 16 dinucleotides of CSFV.

Taking into view the abundant dinucleotide frequencies which affects the usage of codons, none of the dinucleotide frequency was equivalent to the estimated theoretic value (=1.0), indicating that the dinucleotides frequencies values were varying. Among all the 16 dinucleotides, the frequency of dinucleotide CG (0.430) was underrepresented (≤0.78) whereas the frequencies of CC (1.250) and TG (1.240) were overrepresented (≥1.23), CT (1.209) was marginally overrepresented. The results show that the usage of codons was subjected to the abundant dinucleotide frequencies (Figure-6).

## Discussion

In the present study, codon usage bias was analyzed using 115 complete coding sequences of CSFV, including one sequence from our laboratory. RNA viruses have their mutation rates higher and these rates are associated with the evolution and virulence factors to get adapted to the host environment. The mutation pressure, natural selection, frequencies of the mononucleotides, and G/C content are the factors that are associated with the evolution of viruses and the usage of codons. The evolution of the virus is usually determined by the mononucleotides at the third codon position. The codon usage pattern usually tends to get affected by the varying nucleotide arrangement in the genome [5-10,26,31].

In this study, most of the codons in CSFV ORFs were found to be ending with G or C. Mononucleotide A (31.27%) and A_3 (_28.00%) at the third position were found to be higher compared to the other nucleotides in CSFV. The GC_3_ content was 0.46 which shows a small variation compared to GC_1_ and GC_2_. The variations in the base nucleotides and GC contents showed that there are mutations in the CSFV genome.

The RSCU values of 59 codons were calculated for 115 CSFVs and the analysis indicated that the codons ending with G or C were abundant than those of A or C ending codons. The ATA, AGA, AGG, and CTG were overrepresented and ACG, CGT, CTT, and GCG were found to be underrepresented. Seventeen codons were found to have codon usage bias. AGG (2.86) was more overrepresented and GCT (0.09) was more underrepresented, whereas in [4] the AGG and GCT of CSFV were found to have 2.76 and 0.12, respectively, indicating that there is less number of mutations. The variations among the synonymous codons were visualized by plotting the PCA plot which showed variation among the codons and are visible in the graph. Each axis in the PCA was 59 synonymous codons and the points in the PCA plot were the number of coding sequences, that is, 115 sequences used in this study.

The ENC values are very essential to obtain the codon usage bias and are considered to be very significant in codon usage analysis. The average ENC value was found to be 52.69±0.47, indicating the low codon preference and a minimum bias of the codons. The mean ENC value for Atypical Porcine Pestivirus (APPV) was 54.62±0.09 [8], Porcine Astrovirus (PAstV) was 53.89±1.90 [10], and CSFV was 51.85±0.39 obtained using 76 complete CSFV genomes [4]. On comparing with the above values, ENC values in the present study showed 52.69±0.47 which is low for APPV and PAstV and high for CSFV [4]. It is conclusive that the overall codon usage bias is moderately less in this study. The ENC plot displays GC_3_ values against ENC values revealing the bias in usage of synonymous codons in CSFV. In the ENC plot, each point of ENC-GC_3_ is found lying below the expected ENC curve, indicating that the codon usage pattern was shaped by the mutation combined with natural selection. Although the ENC plot showed bias in the codon usage which was not so precise; hence, the analysis of the neutrality plot was carried out. This indicated that the rate of mutations in RNA viruses is significantly high.

The CAI values indicated that the nucleotide compositions and mutation pressure are the important factors affecting the codon usage pattern. The CAI values ranged from zero to one; the higher values (closer to 1) indicate that the usage of codons is similar and lower values indicate that the usage of codons is dissimilar (closer to 0). The CAI in this study was 0.71, which revealed that there was a similarity in the codon usage.

In the analysis of the neutrality plot, the GC_12_ and GC_3_ correlated significantly, which infers that mutation pressure plays a significant role in the codon usage bias when compared to natural selection. Obtained R-squared value of 0.4905 and p< 2.2e^-16^ showing that the plot is substantial. The PR2 plot was plotted using the obtained values with A3/(A3+T3) as ordinate and G3/(G3+C3) as abscissa [8]. It is seen that there is a codon usage bias visualizing the PR2 plot. The nucleotide G is not equal to nucleotide C (G≠C) and as nucleotide A is not equal to nucleotide T (A ≠T), if there is no bias then nucleotide A is equal to nucleotide T (A=T) and nucleotide G will be equal to nucleotide C (G=C) [10,29]. The analysis showed a codon usage inequity between AT and GC at the third codon base position, indicating that in addition to the mutation, the natural selection and/or good adaptation of the virus in pig population (hypothetically) might have affected the patterns of codon usage in CSFV [32,33].

The frequencies of dinucleotides are affected by selection, mutation, and usage of the codons. In this study, the abundance of frequencies of 16 dinucleotides was obtained and plotted with frequencies as ordinate and the dinucleotides as abscissa. The colors in the graph were to differentiate the 16 different dinucleotides. The dinucleotide CG is underrepresented and dinucleotide TG and CC are overrepresented due to natural selection. The dinucleotide CG is usually underrepresented in most of the viruses [30,31,34,35]. The knowledge on codon usage bias in 115 CSFV genome obtained in this study would be of much needed in designing marker vaccine and vaccinology for CSF.

## Conclusion

The synonymous codon usage of 115 complete coding sequences of CSFV has been analyzed. In the present study, it was observed that the codon usage pattern is directly influenced by compositions of mononucleotides, frequencies of dinucleotides, and GC content in CSFV. The study reveals that the evolution of the CSF virus was driven by the mutations in the codons. Evolutionary forces driving the evolution and diversity of CSFV is poorly understood. There are scanty reports on such studies using field isolates. It was shown in Cuban pig population that the vaccination under control program has led to positive selection on B/C domain of the E2 protein for viral isolates circulating in Cuba (subgenotype 1.4) [36]. It was found that vaccination could affect CSFV diversity and might lead to the evasion of the immune response through recombination and point mutation, influencing the population dynamics, evolutionary rates, and adaptive evolution of CSFV [37,38]. Therefore, it is also possible that CSF viruses/strains while evading host immune mechanisms undergo evolution and diversity through recombination and point mutations. The present study undertaken was more focused on codon usage analysis using nucleotides and hence did not comment much on other methods of evolution. The analysis using various methods to study the codon usage bias of CSFV has been explained. Preferably, the codon usage bias observed here is due to the mutation in the nucleotides. The synonymous codon usage pattern and the dinucleotide frequencies are unique in CSFV. Hence, the evolution of CSFV might be due to mutation pressure combined with natural selection. To the best of our knowledge, this is the first report on codon usage bias and analysis of a large number of CSFV sequences, including the Indian strain of CSFV. Natural selection and mutation pressure are the main factors that influence the codon usage pattern. The information gained from this study will help researchers, academicians, and policymakers to apply such methodologies to various other livestock disease virus strains concerning to marker vaccines and vaccinology to study the evolution and codon usage of various viruses and their genetic evolution.

## Data availability

Supplementary data can be available from the corresponding author on request.

## Author’s Contribution

SSP and KPS: Conceptualized and designed the study. SSP, KPS, and UBI: Conducted the analyses and interpreted the results. UBI and KPS: Drafted the manuscript. BRS: Edited the manuscript. All authors revised, edited, read, and approved the manuscript.
